# Our Daughters—Ourselves: Evaluating the Impact of Paired Cervical Cancer Screening of Mothers with HPV Vaccination for Daughters to Improve HPV Vaccine Coverage in Bamako, Mali

**DOI:** 10.3390/vaccines12091019

**Published:** 2024-09-06

**Authors:** Tiffani Crippin, Karamoko Tounkara, Hayley Munir, Eliza Squibb, Caroline Piotrowski, Ousmane A. Koita, Ibrahima Teguété, Anne S. De Groot

**Affiliations:** 1 GAIA Vaccine Foundation, Providence, RI 02909, USAdr.annie.degroot@gmail.com (A.S.D.G.); 2 GAIA Vaccine Foundation, Bamako 999053, Mali; 3 Criminal Justice Sciences Faculty, Illinois State University, Normal, IL 61761, USA; 4 Department of Infectious Diseases, University of Georgia, Athens, GA 30602, USA; 5 Laboratory of Applied Molecular Biology, University of Sciences, Techniques, and Technologies, Bamako 999053, Mali; 6 Gabriel Touré Teaching Hospital, Bamako 999053, Mali

**Keywords:** HPV, Mali, cervical cancer screening, out-of-school girls

## Abstract

Cervical cancer (CC) is the second most common cancer in Western Africa, accounting for 12,000 cases and 6000 deaths annually. While vaccination against human papilloma virus (HPV) and CC screenings reduce the incidence and mortality of CC in many developed countries, 90% of CC deaths are in low-income countries. Lack of knowledge about the connection between HPV and CC, lack of access to vaccines and screenings, weak healthcare infrastructure, and stigma related to sexually transmitted diseases are among the factors that contribute to this disparity. Previously, we evaluated the knowledge of HPV and CC in Bamako, Mali, showing that knowledge of the link between HPV and CC was very low (less than 8% of participants) and that less than 3% of women were screened for CC. Subsequent implementation of a community-based education program and support for local clinics resulted in a five-fold increase in CC screening at the five participating clinics in 2015. In this study, we paired CC screenings of mothers with HPV vaccination of their daughters to target out-of-school (OOS) girls whom school-based vaccination campaigns would not reach. Our campaign resulted in a 10.7% increase in HPV vaccination.

## 1. Introduction

Cervical cancer (CC) is the most common form of cancer in Malian women with an age-standardized incidence rate of 43.1 per 100,000 women [1,2,3]. Early detection and treatment substantially decrease the incidence and mortality of CC [4]. Following a successful CC screening pilot study in 2001, the Malian Minister of Health recommended that CC screening be available in all health facilities throughout the county in 2010 [5]. However, a 2012 report showed that less than 3% of women surveyed in the capital city of Bamako had ever been screened for CC, and by 2015, only 15.6% of women reported being screened [6,7]. Low screening rates in the capital suggest that little to no screening is occurring at rural sites, as screening coverage in rural Sub-Saharan Africa is generally lower than in urban areas [8]. In 2022, the World Health Organization reported 2436 new CC diagnoses and 1431 CC deaths; however, only 5% of women aged 35–49 were screened from 2017 to 2022 [3,9].

Human papilloma virus (HPV) is one of the most common sexually transmitted infections worldwide, affecting around 300 million women worldwide [10,11]. Over 170 HPV genotypes have been identified globally, with 13 strains designated as high-risk HPV (HR-HPV) due to their carcinogenic potential [12,13,14]. Of the HR-HPV strains, HPV 16 and HPV 18 are reported in roughly 70% of invasive CC cases globally [15]. In a study of unscreened Malian women between 15 and 65, HPV 16 or 18 was found in 12% of the 202 participants with subsequent visual inspection identifying 2.5% with suspicious cervical abnormalities [16,17]. Among women with normal cytology, 4% were found to have HPV 16/18 [2]. Overall, HPV 16 and 18 were found in 82.5% of CC cases in 2017 and 51% in 2021 [2,18].

Cervarix**^®^**, Gardasil**^®^**, and Gardrasil**^®^**9 have been shown to be effective against both HPV 16 and 18, and HPV vaccination and cervical screenings are commonly practiced in developed countries [19,20,21,22]. However, less than 30% of lower- and lower-middle-income countries have introduced HPV vaccination, and only 20% of women in these countries have been screened for CC [23,24]. HPV vaccination is expected to have a significant impact on the CC burden, with predictive modeling suggesting that 90% HPV vaccine coverage in Mali will decrease the relative risk of developing CC by 89% [16].

In our 2011 study examining knowledge, attitudes, and practices related to HPV and CC, we found that less than 8% of the 301 male and female participants recognized a connection between HPV and CC [6,25]. To fill this knowledge gap, in 2014, the GAIA Vaccine Foundation (GAIA VF) carried out an education and awareness campaign by engaging local healthcare providers and using a storytelling cloth that visually depicted the connection between HPV and CC [7]. The campaign resulted in a five-fold increase in screening, with over 3000 Malian women seeking screening over a 6-month period.

In 2015, the Malian national and local government authorities on vaccination (National Center for Immunization and Regional Department of Health, respectively) conducted a Global Alliance for Vaccines and Immunization (GAVI)-funded school-based HPV vaccination pilot in Bamako. However, school-based vaccination programs in Mali were expected to exclude the 51.2% of adolescent girls reported to be “out-of-school [26].” By 2017, there were still about 15,000 doses of the quadrivalent vaccine, Gardasil**^®^**, expected to expire in 2018 and 2019. These doses were offered to the GAIA Vaccine Foundation to perform a study in Commune I that would evaluate the efficacy of pairing mothers’ cervical cancer screenings with daughters’ HPV vaccinations.

In partnership with the Malian government, GAIA VF launched the “Our Daughters, Ourselves” campaign in 2018, offering community-centered education sessions as well as free CC screenings and HPV vaccinations for mothers and daughters. There were three main objectives for the campaign and study. The first was to use GAIA VF’s storytelling cloth approach to organize targeted HPV vaccination outreach by clinicians and community health workers to identify mothers who have unvaccinated OOS daughters in six community clinics and the surrounding neighborhoods in Commune 1 of Bamako, Mali. The second was to provide technical assistance and support to six clinics to perform paired CC screening and CC treatment for mothers with the HPV vaccination for their OOS daughters. Finally, GAIA VF aimed to work with local health authorities to measure the impact of the “Our Daughters, Ourselves” intervention on HPV vaccination coverage in Commune 1, as compared to other nearby locations where the storytelling cloth intervention did not take place.

## 2. Materials and Methods

### 2.1. Collaborative Development of the HPV/CC Targeted Campaign and the Design of the Cloth

**Figure 1:**
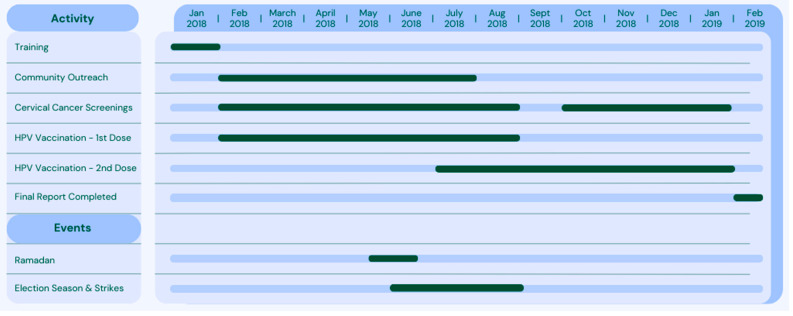
Project Timeline.

Figure 1 shows a timeline of events for this project. A community-led education campaign was launched on 1 February 2018, in Commune 1, Bamako, Mali. The campaign was a collaborative effort between GAIA VF, University of Sciences, Techniques and Technologies of Bamako (UTTSB), and the Malian government to raise awareness of HPV and CC, increase rates of CC screening, and increase vaccination of girls, particularly those that would be missed by school-based national vaccination campaigns. Funding was provided by the Investigator-Initiated Studies Program of Merck Sharp & Dohme Corp (MISP #56611).

Community health educators provided education sessions in clinics and neighborhoods using the storytelling cloth approach pioneered in our 2015 campaign, and educational radio announcements advertising the HPV vaccine and cervical cancer screening ran in February 2018 and August 2018 [7]. Five community health clinics and the district’s Health Reference Center provided free CC screenings and vaccinations. Knowledge of HPV and CC was assessed via survey, as was the effectiveness of the educational campaign in getting women into the clinics for screening.

The survey design, development, and protocol were completed by GAIA Vaccine Foundation staff. The protocol design, surveys, and consent forms were reviewed by a US-based institutional review board (Ethical and Independent Review Services) on 16 October 2017 (17106-01) and by the University of Bamako ethics review board. The objectives, benefits, and risks of participating in the study were explained to the patients at the community clinics and Health Reference Center in Bambara or French. Informed consent was obtained, as per approved protocol. The 3-page survey is provided in Supplementary Materials.

**Figure 2:**
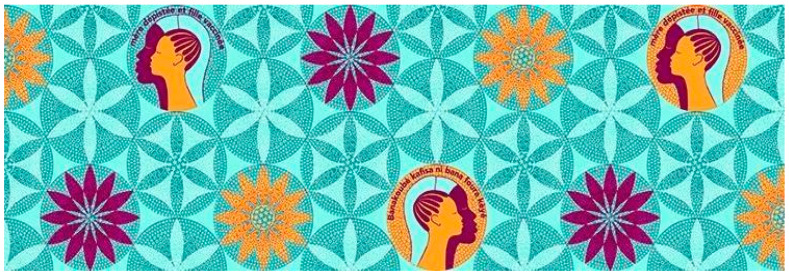
Storytelling Cloth.

The storytelling cloth used for the “Our Daughters, Ourselves” program (Figure 2), designed by Eliza Squibb, depicts geometric flowering patterns based on mathematical multiples of 6 and 12 petals. Both numbers serve as medical appointment reminders built into the pattern. According to Malian National protocol at the time, girls needed the second dose of the vaccine 6 months after the first dose, and women needed follow-up CC screening 12 months after a normal screen. Patterns of healthy cells that transform to cancerous cells when they encounter an HPV virus create a subtle watermark across the floral geometry. The logo for the program contains a woman’s profile and a girl’s profile encircled with the Malian proverb, “It is better to prevent than to cure”, and the French slogan, “Screened Mothers and Vaccinated Daughters”. The pattern was produced by the Malian textile company COMATEX (Ségou, Mali) and printed in two different colorways to offer multiple style options to health workers.

### 2.2. Training Community Partners and Outreach Sessions

GAIA VF hosted a 2-day training on the HPV vaccine and its potential to prevent CC. Eight community health workers from each of the five participating clinics attended the training to ensure the effectiveness of neighborhood outreach. Community health workers were given the storytelling cloth to use as a visual to teach community participants about the HPV vaccine and how it prevents CC. Community health workers also informed participants about the eligibility requirements for the HPV vaccine and where to receive a free CC screening in their community.

Three healthcare workers from each community clinic participated in a separate training on outreach methods and survey protocols. Nurses and midwives conducted weekly education sessions in the waiting areas of each community clinic and surveyed consenting mothers. Since the sessions were conducted in the waiting areas, everyone visiting the clinic that day overheard the outreach. Mothers and daughters who were visiting to participate in the project were reminded of the importance of CC screening and HPV vaccination, and women learning about the project for the first time were encouraged to revisit the clinic with their daughters for free CC screening.

Many women forego medical exams, like CC screening, without already presenting with symptoms due to the cost. To overcome this barrier, we provided women with discount cards that covered both the costs of the CC screening and the clinic admission fees during the outreach sessions. By combining neighborhood outreach and clinic activities with subsidized clinic costs, we ensured that difficult-to-reach women and girls would also be included in the project.

### 2.3. Screening and Vaccination Training and Support for Healthcare Personnel

Each of the six community clinics received technical and material assistance from GAIA VF to help ensure that CC screening and HPV vaccination goals were met. The HPV vaccine was provided by the National Center for Immunization, and the Regional Department of Health hosted a 6-day training for healthcare workers to perform CC screening by visual inspection with acetic acid and Lugol’s iodine (VIA/VILI) and deliver the HPV vaccine. Clinic personnel (one doctor, one midwife, two nurses, and three community health workers per site) also received a small honorarium (about $100 per doctor, $80 per midwife, $60 per nurse, and $40 per community health worker) to participate in the project. Women made up 95% of the participating healthcare providers and community health workers.

#### Protocol for Engaging with Patients

**Figure 3:**
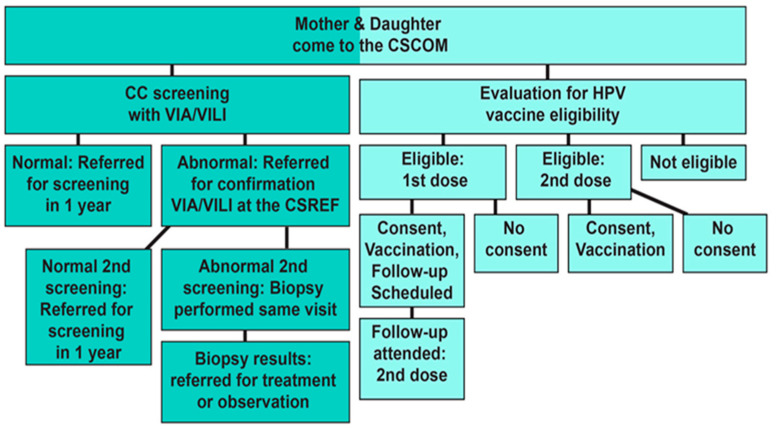
Protocol Flow Chart.

Figure 3 outlines the project protocol. When a mother-daughter pair arrived at the clinic, trained clinic staff explained and proposed the study. If the mother had a discount card (obtained by previously attending an education session), the clinic waived the cost of the CC screening. Daughters who accompanied their mothers to the clinic participated in an education session about the HPV vaccine while their mothers received a CC screening.

Mothers with normal cytology were scheduled for a follow-up screening appointment the next year, per the Malian national health protocol at the time. When a mother received a positive CC screening, she was directed to the district’s Health Reference Center for a confirmation screening. If the second screening was also positive, the mother was given a biopsy during that same appointment. This biopsy was delivered to the National Laboratory for analysis, and the results were sent to the local attending physician within two weeks. Confirmed cases of precancerous and cancerous lesions were referred to the OB/GYN department of the Gabriel Touré Teaching Hospital for observation and treatment, including cryotherapy or a loop electrosurgical excision procedure to remove precancerous lesions. Quarterly supervision, abnormal pathology review, and follow-up rates were examined for each site by the gynecological surgeon and expert for the campaign, Dr. Ibrahima Téguété at Gabriel Touré Teaching Hospital.

Informed consent was obtained from the mother and/or from the father by phone (a method that was approved by the local ethics committee). Mothers and daughters were interviewed to determine if the girls had already received the vaccine at school. All eligible and consenting girls were vaccinated with Gardasil while their mothers were screened. Vaccination cards, which are provided to the parents of children following vaccination, were reviewed. Mothers were contacted 6 months later, by phone call, to bring their daughters for the second dose of the vaccine.

### 2.4. Data Collection and Statistics

Data collection and compilation from each clinic were executed by GAIA VF’s Malian team and supervised by Dr. Karamoko Tounkara (pediatrician and GAIA VF’s National Director) and Dr. Ousmane Koita (Director of the Laboratory of Applied Molecular Biology). The team recorded (1) the number of girls vaccinated, (2) the number of girls returning for the second dose of the vaccine, and (3) the number of mothers who received CC screening. Vaccination rates were provided to the local public health authorities, and data from Commune 5 and historical Commune 1 data were obtained from the Regional Department of Health.

A total of 511 women who visited the clinic for free CC screening also participated in verbally administered surveys to evaluate (1) satisfaction with the mother-daughter program, (2) the impact of the storytelling cloth, and (3) whether we were reaching OOS girls. The survey was administered by a nurse trained to administer surveys orally, as many women in the study group are illiterate. Raw data were collected on survey forms and coded by hand into Microsoft Excel by a trained data input technician. The study was case controlled but not blinded.

## 3. Results

Table 1 provides descriptive statistics from the survey. The average age of women participating in the survey was 34 years old. Only 15.5% (N = 79/511) of the women had ever been screened for CC, and only 59% had heard of CC before arriving at the clinic. The 511 survey participants brought 727 girls with them to the clinic. The average age of the girls was 9, and most of them attended privately run or informal schools.

Over the course of 6 months, GAIA VF conducted educational outreach sessions in the community aiming to increase demand for CC screening and HPV vaccination. Our goal was to reach 7000 mother-daughter pairs. We expected 100% of women to accept screening, 5% of them to screen positive, and 46% of the initial positive screenings to be confirmed at the hospital, leaving 161 women to receive treatment. We expected 70% of eligible daughters to have already received HPV vaccination in school, leaving around 2100 vaccine-eligible girls in our sample.

The project launched in February 2018 and ran through January 2019. The original protocol outlined that we would offer free CC screenings while distributing the first dose of the HPV vaccine, but free CC screenings would not be available with the second dose. After ending the free screenings in September 2018, it became more difficult to get girls to return for the second dose. As such, we reinstated the free screenings in October to ensure that we fully vaccinated as many girls as possible. The five community clinics averaged 87.6 screenings per month, and the Health Reference Center averaged 123 (Supplemental Figure S1A). A total of 5971 CC screenings were performed (85.3% of our expected value of 7000). CC screening was accepted by 99.2% of women. Less than 1% (0.095% N = 57/5971) screened positive for CC, and only 37% (N = 21/57) of the women who originally screened positively also received a positive biopsy (Supplemental Figure S1B). All 21 women with a positive biopsy received surgical excision of the cancer.

From February to August, we vaccinated 793 girls with the first dose of the HPV vaccine. Then, from July to January 2019, we vaccinated 749 of those girls with the second dose. According to data from the National Center for Immunization, in 2017, the national school-based HPV vaccination campaign fully vaccinated 7029 girls in Commune 1. Our campaign fully vaccinated another 749 girls, leading to a 10.7% increase in overall vaccination completion (Supplemental Figure S2A,B).

**Figure 4:**
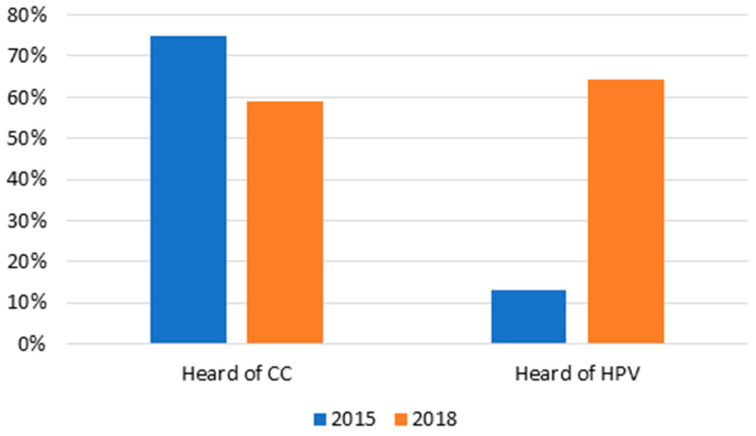
CC and HPV Knowledge by Year.

Figure 4 compares the results of a prior study in 2015 to the survey results from this study. In 2015, knowledge of CC increased dramatically to over 70%, yet in this campaign, less than 60% of surveyed women had heard of CC [7]. This may be attributed to survey execution. In the 2015 study, women were surveyed throughout the duration of the study, while in 2018, researchers only surveyed the first 500 women to present for CC screenings. Despite this, we observed a significant increase in the number of surveyed women who had heard about HPV. Knowledge about HPV increased from 13% during our first campaign in 2015 to 64% (N = 329/511) in 2018.

In addition to the increase in knowledge about HPV, nearly 75% of women surveyed for this project reported that the education sessions (either in the clinic or in the neighborhood) were the reason that they participated in the paired intervention (N = 293/394).

Most women who came into the clinic were able to give consent for their daughters to be vaccinated without receiving permission from the father. Only 8.2% (N = 42/511) of women said the child’s father would be the only one able to give consent for her to be vaccinated. This is a dramatic shift from our 2015 finding that 52% of women would need to contact their husbands to give consent.

Lastly, we asked all women visiting the clinics about the children in their home to get a better idea of where to find OOS girls for vaccination. According to our survey, 45% (N = 329/727) of girls in Commune 1 of Bamako attended privately run or informal schools, while only 15% (N = 111/727) attended publicly run schools. Interestingly, only 8% (N = 57/727) of women surveyed said that their daughters worked and did not attend school. However, 28% (N = 205/727) did not disclose the activities of their daughters.

**Figure 5:**
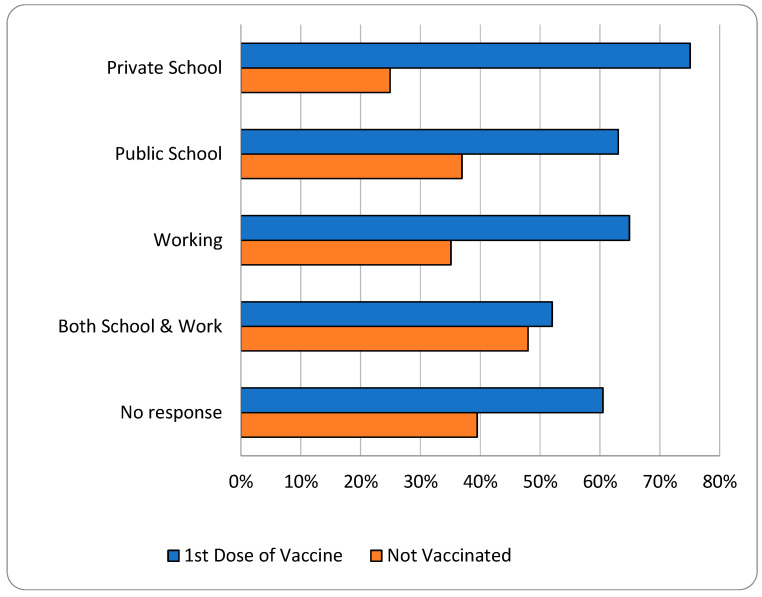
Girls’ Activities by Vaccination Status.

Figure 5 shows how many girls from each group were reached by the government-led vaccine campaign. According to our survey, the government campaign reached 75% (N = 247/329) of girls that attended a privately run school, 63% of girls who attended publicly run schools (N = 70/111), 65% of working girls (N = 37/57), 52% of girls who attended school and worked (N = 13/25), and 60% of girls whose activities were not disclosed.

## 4. Discussion

**Figure 6:**
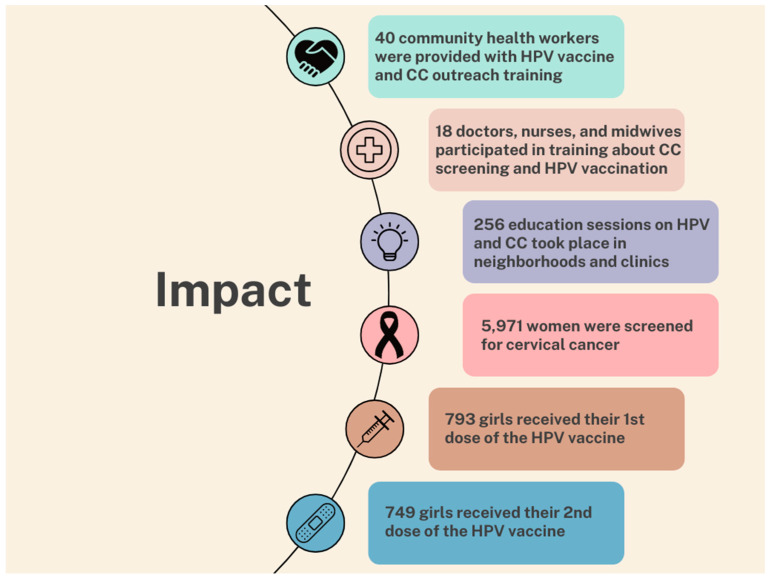
Impact of the Project.

Figure 6 shows the full impact of the $224,100 spent on this project. The figure explains the impact of the project on training, CC screenings, and HPV vaccination. Discount cards for screenings cost only $2 per mother, and screening supplies cost only $4 per mother. Vaccination supplies cost only $6 per girl. The results of this study suggest that a small investment and pairing mothers’ CC screenings with daughters’ HPV vaccinations is an effective strategy for reaching girls for vaccination who would otherwise be missed. This strategy allowed us to fully vaccinate an additional 749 girls who were missed in the school-based vaccination program either because they did not attend school or went to a private school that was not included in the government campaign. In addition, 793 girls received one dose, which has been shown to be sufficient for durable protection [27,28,29]. While vaccinating boys would also help to improve coverage in the future, as of 2024, Mali still limits vaccination to girls.

The effectiveness of the community education campaign is observed through the increase in the surveyed mothers’ understanding of the link between CC and HPV. Specifically, understanding of the link between CC and HPV in our 2015 study was 45% (N = 226/500). In the 2018 study that paired mothers’ CC screenings with daughters’ HPV vaccinations, understanding of the link between CC and HPV increased to 64% (N = 329/511). In addition to increasing mothers’ understanding of the connection between CC and HPV, pairing the services reduces the costs of visiting the clinic (e.g., taking time off work/school, finding childcare for other children, etc.). Moving forward, the incorporation of self-sampling and testing for HPV could provide another effective, low-cost means of engaging communities and motivating women to get a CC screening who might otherwise initially decline more invasive procedures, such as a pap smear [30].

**Figure 7:**
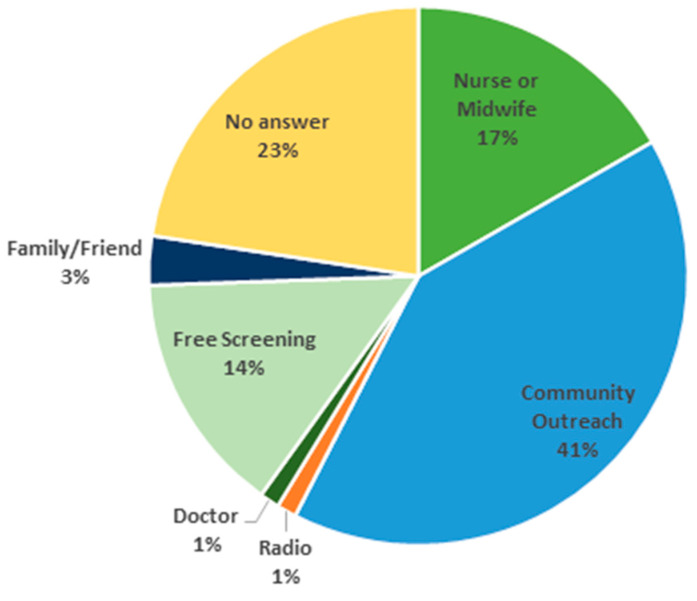
What prompted you to visit the clinic today?

Figure 7 shows the breakdown of reasons that surveyed women gave for visiting the clinic. Consistent with the survey from 2015, in 2018, surveyed women listed community outreach as the primary reason for visiting the clinic. However, in 2015, only 14% of women were motivated to seek CC screening through community outreach, but in 2018, 41% of women reported community outreach as a reason for their visit. This project offered free CC screenings to mothers who brought their daughters to the clinic. The survey indicates that 14% of mothers said that the free screening was their primary reason for visiting the clinic. In this project, a total of 5971 women were screened for CC, and 793 girls received the first dose of the HPV vaccine. All mothers received a free screening even if their daughter was ineligible for the vaccine due to age or because they had already received the HPV vaccine.

One concerning feature of the 2018 project is the decrease in knowledge of CC among the women surveyed. Figure 6 illustrates a decrease in CC knowledge from 2015 to 2018. We interpret this finding to mean that while community outreach is effective, continued education is important to help the target population retain the information and ensure that we reach new audiences. In other words, community outreach should be paired with efforts to educate the population to increase awareness of both HPV and CC. As noted above, it is also important to keep in mind differences in survey administration when considering the decline in knowledge of CC. In the 2015 study, participants were surveyed throughout the study, and in 2018, only the first 500 participants were surveyed.

The current project faced several challenges that need further discussion. In 2015, 87% of women wanted their child to be vaccinated against HPV/CC. However, only 33% of mothers answered that they could unilaterally give consent for their daughter to be vaccinated. This was a major concern during the planning for the Mothers-Daughters campaign, as it presented a possible complication for vaccinating daughters at the same appointment where their mothers received a CC screening. The concern was that if a father was not present to consent to the HPV vaccination, the daughters would not be vaccinated. Interestingly, once the vaccine was readily available in 2018, many mothers were able to consent to their daughters’ vaccination without even calling the father. In situations when a father’s consent was necessary, a phone call was adequate. Therefore, this was not a major issue facing the project.

Another challenge facing this project was the political climate. In 2018, public health activities in Bamako were negatively impacted by the political protests and healthcare worker strikes that took place during July, the month of the presidential election. The opposition coordinated massive protests throughout Bamako, which were often met with counter-maneuvers and repression by the government [31,32]. After losing the election, the opposition continued to hold protests, challenge election results, and fight for increased transparency and security from the ruling party [33,34]. This heightened level of political turmoil, combined with clinic strikes, decreased opportunities to get women and girls into clinics for screenings and/or vaccinations. Therefore, this project reached fewer women than we had hoped.

The results of this study are limited to low literacy populations where a strategy focused on a visual and verbal outreach style is most effective. Outreach for high literacy populations should likely take on a different form. It is also important to acknowledge the other factors that could not be controlled in this study: the effects of the government campaign (including radio announcements), the occurrence of Ramadan in September 2018, and many women bringing in daughters who had already been vaccinated or were too young to be vaccinated. Despite these challenges, the present study demonstrates a low-cost, culturally relevant intervention style that undoubtedly contributed at least in part to vaccinating an additional 10.7% of girls in Commune I during Mali’s HPV pilot with GAVI. The cost of CC screening is about $2 per woman. By waiving that cost for 5971 women, we were able to fully protect 743 girls against CC in the future. This type of intervention has the potential to be scaled up to accompany the launch of the HPV vaccine expected in many countries in 2024 and 2025.

## 5. Conclusions

There are three major conclusions to be drawn from this study. First, linking CC screening to HPV vaccination improved uptake of the HPV vaccine for an additional 10.7% of girls. Most women visited the clinic after taking part in an education session, demonstrating that outreach does motivate women to seek care. If the goal is to eradicate HPV transmission, then combining this approach with school-based vaccinations could help achieve this goal.

Second, knowledge of HPV increased from 13% during our first campaign to 64% during the current campaign. This suggests that the outreach method presented here more effectively transmitted information linking CC to HPV to participants than our previous campaign. Our previous campaign focused on HPV vaccination as a future idea rather than a currently available vaccine. It is important to note that ongoing national campaigns on HPV vaccination and the actual availability of the HPV vaccine may have also impacted knowledge of HPV.

Finally, this study demonstrates that education helps motivate women to seek care. Interest in CC screening has increased, even among younger women. As a result, the overall prevalence of CC was much lower in this cohort than was recorded during our 2015 intervention. In our study, most women mentioned having received community outreach as the motivation for attending clinic. The development of HPV self-testing may also increase the number of women willing to accept screening, as women who are no longer sexually active are less inclined to accept the more invasive cervical cancer screening with VIA/VILI. Modification of the dosing schedule to one dose, with two doses for immunocompromised girls, will also facilitate vaccine uptake and completion. The “Our Daughters, Ourselves” project explicitly linked HPV and CC by connecting mothers and daughters. For example, the campaign stated that “daughters should be vaccinated, and mothers should be screened”. Taken together, the results indicate that pairing CC screenings of mothers with HPV vaccinations for their OOS daughters is a meaningful approach to increasing HPV vaccination rates in OOS girls who would otherwise not come in contact with school-based vaccination programs.

## Figures and Tables

**Table 1:** Descriptive Statistics.

Woman Age	34 Years Old
Women previously screened for CC	79 (15.5%)
Women who heard of CC before today	301 (59%)
Number of girls brought to clinic	727
Girls receiving first dose of vaccine	491 (67.5%)
Average age of child	9 years old
Girls attending public school	111 (15%)
Girls attending private school	329 (45%)
Girls working	57 (8%)
Girls going to school and working	25 (3%)
Choose not to answer	205 (28%)

## Data Availability

The raw data supporting the conclusions of this article will be made available by the authors.

## Supplementary Materials

The following supporting information can be downloaded at https://www.mdpi.com/article/10.3390/vaccines12091019/s1, Figure S1: (**A**) Cervical Cancer Screenings by Month; (**B**) Biopsies Performed and Positive; Figure S2: (**A**) First Dose of HPV Vaccine by Clinic, (**B**) Second Dose of HPV Vaccine by Clinic.
