# Supplementary material for: Our Daughters—Ourselves: Evaluating the Impact of Paired Cervical Cancer Screening of Mothers with HPV Vaccination for Daughters to Improve HPV Vaccine Coverage in Bamako, Mali
Source: Vaccines (Basel). 2024 Sep 6;12(9):1019. doi: 10.3390/vaccines12091019 (PMC11435951; doi:10.3390/vaccines12091019)

Supplemental Figures

Figure 1SA: Cervical Cancer Screenings by Month

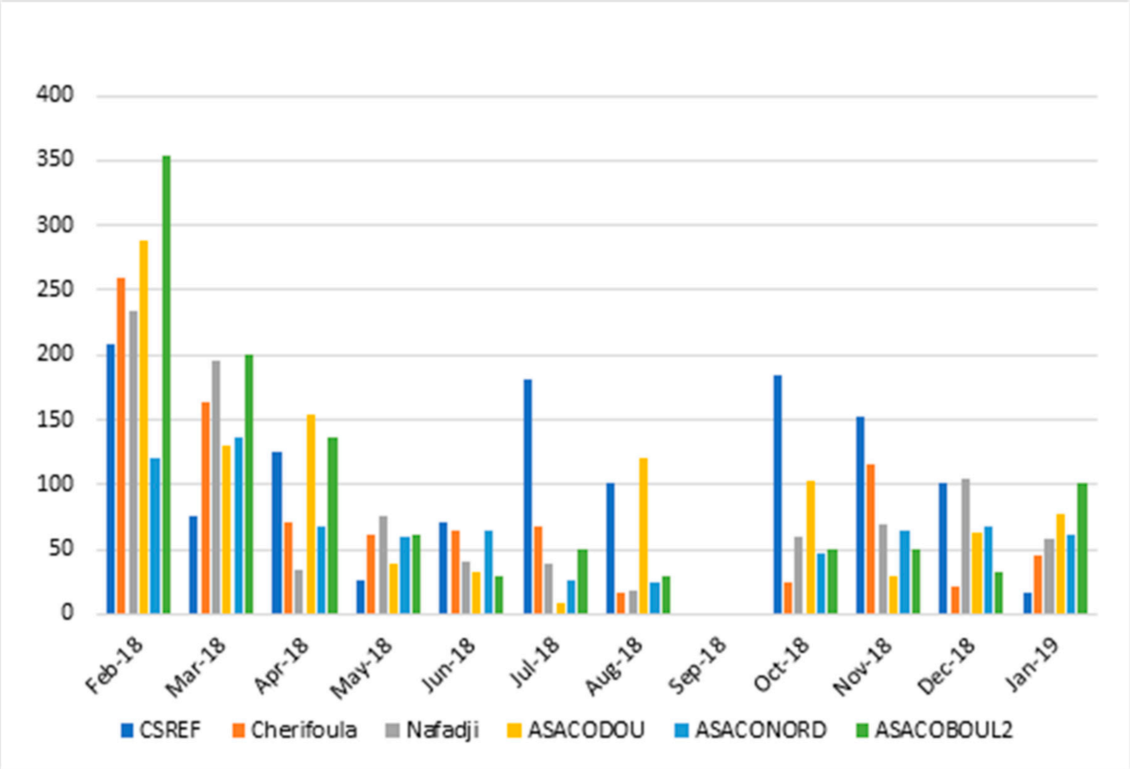

Figure 1SB: Biopsies Performed and Positive

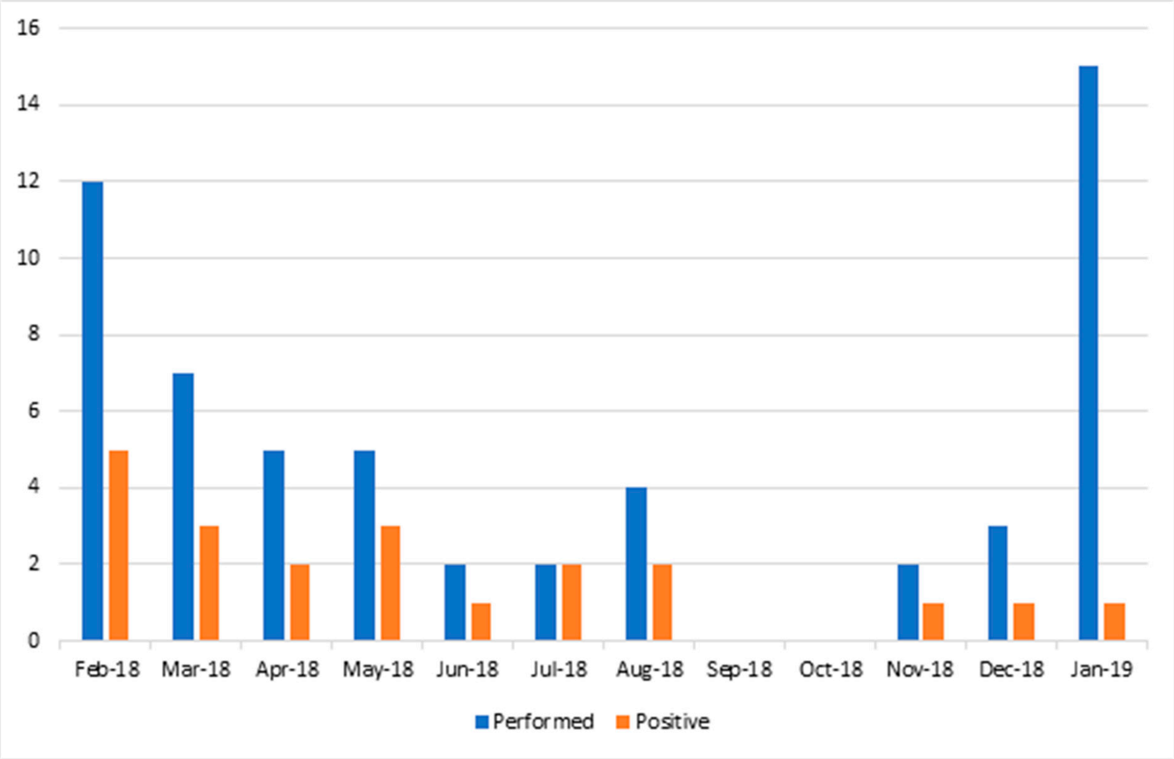

Supplemental Figures

Figure 2SA: First Dose of HPV Vaccine by Clinic

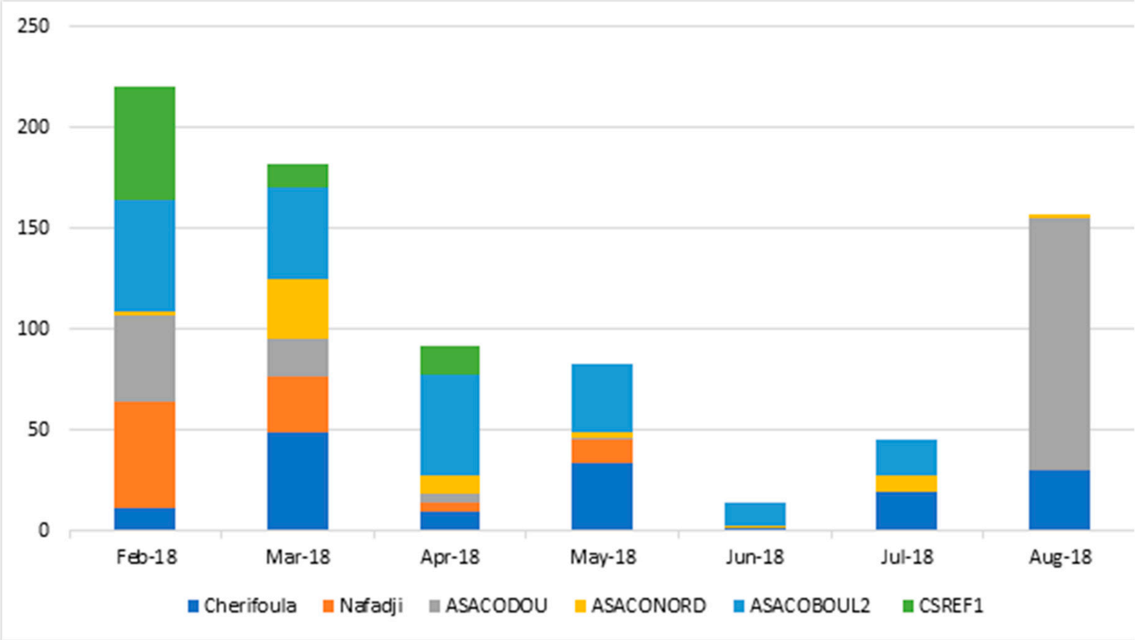

Figure 2SB: Second Dose of HPV Vaccine by Clinic

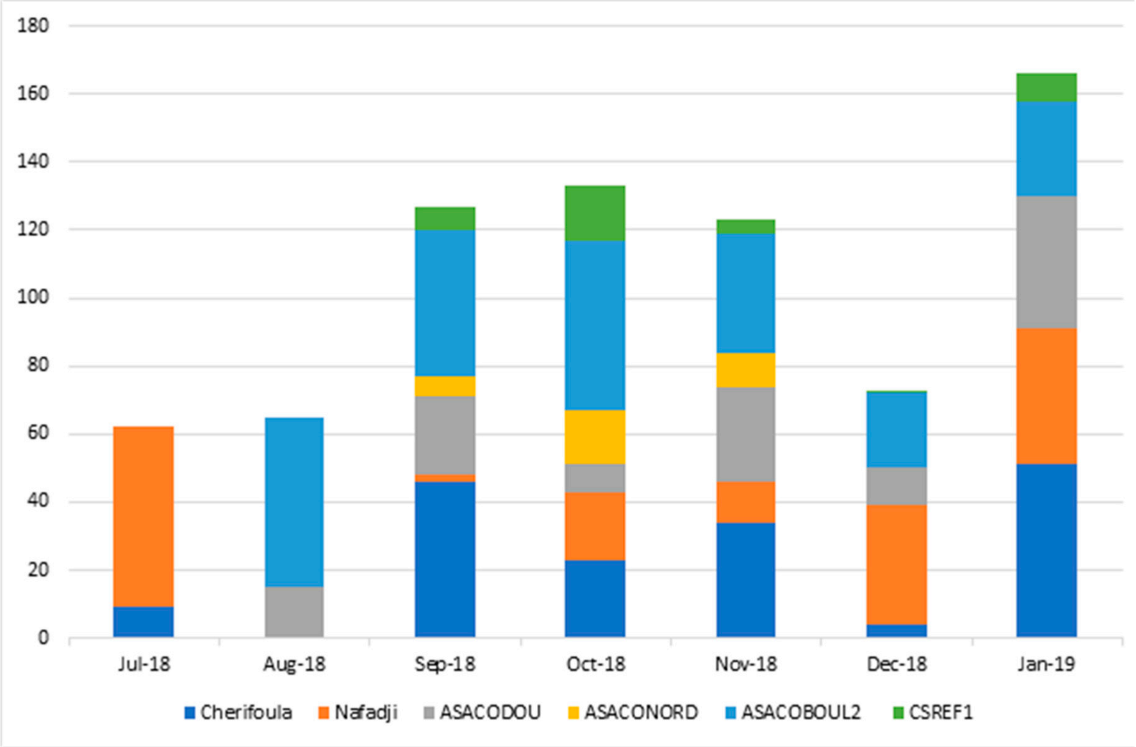

Supplement: Supplementary file 1 [file vaccines-12-01019-s001.zip › vaccines-3144413-supplementary.pdf]
